# A Comprehensive Review of Treatment Strategies for Early Avascular Necrosis

**DOI:** 10.7759/cureus.50510

**Published:** 2023-12-14

**Authors:** Ashutosh Lohiya, Nareshkumar Dhaniwala, Ulhas Dudhekar, Saksham Goyal, Siddharth K Patel

**Affiliations:** 1 Department of Orthopaedics and Traumatology, Jawaharlal Nehru Medical College, Datta Meghe Institute of Higher Education & Research, Wardha, IND; 2 Department of Orthopaedic Surgery, Jawaharlal Nehru Medical College, Datta Meghe Institute of Higher Education & Research, Wardha, IND; 3 Department of Orthopaedics, Jawaharlal Nehru Medical College, Datta Meghe Institute of Higher Education & Research, Wardha, IND

**Keywords:** rehabilitation, multidisciplinary care, emerging therapies, treatment strategies, osteonecrosis, avascular necrosis

## Abstract

Avascular necrosis (AVN), characterised by compromised blood supply leading to bone necrosis, poses a significant challenge in orthopaedic and rheumatologic practice. This review comprehensively examines early AVN treatment strategies, including aetiology and risk factors, clinical presentation, conservative and surgical approaches, emerging therapies, and rehabilitation. Key findings underscore the importance of early detection, personalised treatment plans, and a multidisciplinary approach involving orthopaedic specialists, rheumatologists, and physical therapists. The implications for clinical practice emphasise individualised care, staying abreast of emerging therapies, and patient education. Recommendations for future management strategies highlight the need for imaging technology advancements, regenerative therapies integration, and ongoing research into genetic and molecular pathways. As the field continues to evolve, translating research findings into clinical practice holds promise for improving outcomes and enhancing the overall quality of life for individuals affected by AVN.

## Introduction and background

Avascular necrosis (AVN), also known as osteonecrosis, is a debilitating condition characterized by the death of bone tissue due to a lack of blood supply. This phenomenon predominantly affects weight-bearing joints, such as the hip and knee, and can lead to significant pain, joint dysfunction, and, if left untreated, irreversible damage. This review aims to comprehensively explore treatment strategies specifically focused on the early stages of AVN [1].

AVN involves the compromised blood supply to bone tissue, resulting in ischemia and subsequent cellular death. Typically occurring in joints, this condition manifests as the gradual deterioration of bone structure, ultimately affecting joint function. Understanding the underlying pathophysiology is crucial for developing effective treatment strategies and interventions that can halt or slow the progression of AVN [2].

Early detection of avascular necrosis is paramount for achieving favourable treatment outcomes. The insidious nature of AVN often means that symptoms may not become apparent until the disease has advanced, emphasizing the need for vigilant monitoring and diagnostic measures. Timely intervention during the initial stages offers the best chance to preserve joint integrity, minimize pain, and prevent the need for more invasive procedures like joint replacement [3].

This review will delve into various aspects of early avascular necrosis, including its pathophysiology, aetiology, and risk factors. The clinical presentation of AVN and diagnostic imaging techniques will be explored to provide a comprehensive understanding of how healthcare professionals can identify the condition in its nascent stages. Moreover, the review will thoroughly examine conservative and surgical treatment approaches, encompassing pharmacological interventions, physical therapy, and advanced surgical techniques. Emerging therapies, rehabilitation protocols, and long-term management strategies will also be discussed.

## Review

Aetiology and risk factors

Traumatic Causes

Fractures and dislocations: Traumatic fractures and dislocations pose substantial risks for the development of AVN. These events have the potential to disrupt the blood vessels supplying the affected bone, resulting in ischemia, a condition where blood flow to the bone is compromised. Notably, specific instances such as fractures of the femoral neck or traumatic hip dislocations are well-documented as high-risk scenarios for subsequent AVN. The severity of the injury and the extent of vascular damage play pivotal roles in influencing the likelihood of AVN. For instance, fractures that involve significant displacement or dislocations causing vascular compromise increase the risk substantially. Timely and appropriate management of these fractures is crucial for minimizing the risk of avascular events. Swift interventions, including surgical stabilization or reduction, are paramount in restoring proper alignment and blood flow to the injured area, thereby mitigating the potential for AVN development. To illustrate, studies have shown that prompt reduction and fixation of a displaced femoral neck fracture significantly reduce the incidence of AVN. Additionally, rehabilitation strategies emphasizing early mobilization and joint protection contribute significantly to a comprehensive approach aimed at preventing the progression of AVN [3].

Joint trauma: Injuries directly affecting the joints, such as severe contusions or crush injuries, can have profound implications for the circulation within the joint structures. The vulnerability of joints to avascular changes underscores the critical importance of vigilant monitoring and early intervention following joint trauma. Joint trauma can lead to microvascular damage, compromising blood supply and initiating the cascade of events that may culminate in AVN. Swift recognition of joint trauma, often facilitated by advanced imaging techniques, enables healthcare professionals to assess the extent of injury and determine appropriate interventions. Early measures may include joint aspiration to relieve intra-articular pressure, immobilization to minimize stress on the injured joint, and pharmacological interventions to manage inflammation and pain. The collaborative efforts of orthopaedic specialists and rehabilitation professionals are essential in orchestrating a timely and tailored approach to mitigate the risk of AVN development in the aftermath of joint trauma [4].

Non-Traumatic Causes

Steroid use: Prolonged or high-dose steroid therapy stands as a well-established non-traumatic cause of AVN. Steroids exert their influence by inducing lipid microembolism and intravascular coagulation, which collectively contribute to compromised blood flow to the bone. The risk of AVN is particularly heightened in individuals undergoing extended courses of steroid treatment, such as those with autoimmune disorders or organ transplants. Clinicians must be vigilant in understanding the risks associated with steroid use, recognizing that specific patient populations may be predisposed to AVN. Monitoring patients on long-term steroid therapy, implementing dose reduction strategies when feasible, and considering alternative treatments are essential aspects of mitigating the risk of AVN in this context [5].

Alcohol abuse: Chronic alcohol consumption has been identified as a significant risk factor for AVN, mainly affecting weight-bearing joints such as the hip. The mechanisms through which alcohol contributes to AVN are multifaceted, involving vascular changes, alterations in lipid metabolism, and impaired bone remodelling. The impact of alcohol on blood vessels can lead to decreased blood supply to the bones, initiating a cascade of events culminating in AVN. Clinicians should actively inquire about and consider the history of alcohol abuse in patients presenting with AVN, particularly in cases involving the hip joint. This awareness allows for tailored interventions, including lifestyle modifications and substance abuse counselling, to address the underlying risk factor and potentially halt the progression of AVN [6].

Coagulation disorders: Conditions affecting blood coagulation, such as thrombophilia and hypercoagulable states, significantly elevate the risk of AVN. In individuals with coagulation disorders, an imbalance in blood coagulation factors can lead to microvascular thrombosis, compromising the blood supply to the bone. Thrombotic events within the small vessels supplying bone tissue contribute to the pathogenesis of AVN. Clinicians should maintain a heightened awareness of coagulation disorders when assessing patients at risk for or presenting with AVN. This involves thoroughly investigating the patient's medical history, family history, and appropriate laboratory testing to identify coagulation abnormalities. Timely recognition and management of coagulation disorders are crucial in reducing the risk and severity of AVN in affected individuals [7].

Common Risk Factors

Age and gender: A prominent demographic factor influencing the manifestation of AVN is age, with a typical onset occurring in individuals aged 30-50. Additionally, there is a noteworthy preference for males in the incidence of AVN. Hormonal fluctuations and age-related changes in bone metabolism during this specific life stage may contribute to the heightened susceptibility observed in males and individuals within this age range. Understanding the intersection of hormonal influences and age-related factors is crucial in elucidating the mechanisms that render this demographic group more prone to AVN. Clinicians should consider these demographic characteristics when assessing individuals for potential risk factors and early signs of AVN, enabling proactive interventions and personalized management strategies [8].

Joint loading and weight-bearing: The mechanical stresses associated with weight-bearing joints, notably the hip and knee, play a pivotal role in developing AVN. Daily activities and excessive joint loading increase the risk of compromised blood supply to these weight-bearing joints. Obesity further exacerbates this risk, emphasizing the critical need for weight management in individuals susceptible to AVN. Excessive body weight places additional strain on weight-bearing joints, creating an environment conducive to the development or progression of AVN. Clinicians should prioritize weight management strategies in susceptible individuals, incorporating lifestyle modifications and personalized interventions to alleviate mechanical joint stress. This multifaceted approach addresses both the mechanical and metabolic factors contributing to AVN, promoting joint health and mitigating the risk of its occurrence [9].

Relationship with Systemic Conditions

Autoimmune disorders: Individuals with autoimmune disorders, notably systemic lupus erythematosus (SLE) and rheumatoid arthritis (RA), face an elevated risk of developing AVN. The complex interplay of inflammatory processes underscores the intricate relationship between autoimmune-mediated vascular changes and AVN. In autoimmune conditions, the immune system's aberrant response can lead to vascular compromise, diminishing blood supply to the affected bone. Furthermore, the use of immunosuppressive medications, often employed in the management of autoimmune disorders, contributes to the multifactorial landscape of AVN development. Clinicians should remain vigilant in recognizing and managing AVN in individuals with autoimmune disorders, emphasizing regular monitoring, early detection of symptoms, and a collaborative approach between rheumatologists and orthopaedic specialists to optimize patient outcomes [10].

Hematologic disorders: Hematologic disorders, including sickle cell disease and other conditions affecting blood composition, pose a significant risk for developing AVN. These disorders can lead to vascular occlusion and ischemia, creating a milieu conducive to AVN. Individuals with sickle cell disease, in particular, experience a heightened susceptibility due to the characteristic sickling of RBCs, leading to impaired blood flow. Regular monitoring and early intervention are paramount in managing AVN in patients with underlying hematologic disorders. Clinicians must adopt a proactive approach, conducting thorough assessments and implementing preventative measures to minimize the risk of AVN in individuals with hematologic conditions. This includes collaborative care involving haematologists, orthopaedic specialists, and other relevant healthcare professionals to provide comprehensive and tailored management strategies [11].

Clinical presentation

Signs and Symptoms

Joint pain: The primary and hallmark symptom of AVN is progressive joint pain, typically localized to the affected area. Initially, the pain may manifest as mild and intermittent, often exacerbated by weight-bearing activities. However, as AVN advances, the pain tends to worsen over time, becoming more constant and severe. This pain is indicative of the compromised blood supply and subsequent necrosis of the bone, triggering inflammatory responses and nerve stimulation. Understanding the evolving nature of joint pain in AVN is essential for early detection and timely intervention to alleviate symptoms and prevent further joint damage [12].

Limited range of motion: As AVN progresses, individuals commonly experience a decreased range of motion in the affected joint. This limitation arises from the compromised structural integrity of the bone and surrounding tissues, leading to stiffness and reduced flexibility. The restriction in joint motion can significantly impact daily activities, affecting mobility and overall quality of life. Monitoring changes in the range of motion is a critical clinical indicator of disease progression and guides therapeutic interventions to preserve joint function [13].

Joint stiffness: Stiffness, particularly noticeable after periods of inactivity, is a prevalent complaint in individuals with AVN. Patients often report increased difficulty initiating movement, and morning stiffness is characteristic. The stiffness results from the inflammatory processes associated with AVN and the compromised lubrication within the joint. Recognizing and addressing joint stiffness early during AVN is crucial for implementing targeted interventions, including physical therapy and joint-preserving strategies [14].

Muscle atrophy: Progressive AVN can lead to muscle atrophy around the affected joint. The combination of pain and reduced use of the compromised joint contributes to muscle wasting. Muscle atrophy not only exacerbates weakness but also further compromises joint function. Rehabilitation strategies focusing on muscle strengthening and targeted exercises become integral components of AVN management to counteract atrophy and enhance overall joint stability [15].

Joint instability: In advanced cases, AVN may progress to joint instability, characterized by a sense of the joint giving way or feeling unstable, particularly during weight-bearing activities. Joint instability poses a significant challenge, affecting balance and increasing the risk of falls and injury. Early recognition of signs of joint instability prompts appropriate interventions, including orthopaedic evaluation and consideration of surgical options, to address the underlying structural issues and restore joint stability. This aspect highlights AVN's evolving and dynamic nature, emphasizing the importance of a comprehensive symptom assessment and management approach [16].

Diagnostic Imaging Techniques

X-rays: X-rays serve as the initial and fundamental imaging modality in diagnosing suspected cases of AVN. This non-invasive technique is pivotal for assessing bone structure and detecting advanced stages of AVN. Characteristic findings on X-rays include joint space narrowing, indicative of degenerative changes, subchondral sclerosis marked by increased bone density, and the formation of crescent-shaped areas of necrotic bone known as crescent signs. X-rays play a crucial role in providing an overview of the skeletal anatomy, aiding in the identification and staging of AVN. While particularly valuable in advanced cases, X-rays may not capture early-stage changes, prompting the use of more sensitive imaging modalities for timely diagnosis [17].

MRI: MRI stands out as a susceptible and specific imaging technique, particularly adept at detecting the early stages of AVN. MRI provides detailed images of soft tissues, offering a comprehensive view of changes in bone marrow and the early signs of necrosis. This modality is particularly valuable in cases where early intervention is crucial, allowing clinicians to visualize the extent of involvement and make informed treatment decisions. MRI's superior soft tissue contrast enhances its ability to identify subtle changes in bone structure, making it an essential tool for the early diagnosis and ongoing monitoring of AVN [18].

CT scans: CT scans complement the diagnostic armamentarium for AVN by offering a three-dimensional visualization of the affected joint's structure. While not as sensitive as MRI in detecting early-stage changes, CT scans provide valuable information, especially when precise anatomical details are essential. CT scans benefit surgical planning for joint interventions, offering detailed insights into the spatial relationships between bones and facilitating targeted procedures. The ability of CT scans to capture fine anatomical details makes them a valuable adjunct to X-rays and MRI in the comprehensive assessment and staging of AVN, ensuring a holistic understanding of the condition for optimal clinical management [19].

Staging of AVN

Importance of Staging

Treatment planning: Staging is pivotal in formulating effective treatment plans for individuals with AVN. By categorizing the condition into distinct stages, clinicians can tailor interventions to the specific needs and severity of the disease. In the early stages, conservative measures such as activity modification and pharmacological treatments may be sufficient to alleviate symptoms and slow disease progression. As AVN advances, staging guides clinicians in considering more aggressive interventions, such as core decompression or joint replacement surgery. This staged approach ensures that treatments are appropriately matched to the evolving nature of the condition, optimizing outcomes and preserving joint function [19].

Prognostication: The stage of AVN serves as a crucial factor in prognostication, offering valuable insights into the likely course and outcomes of the condition. Early-stage AVN may have a more favourable prognosis when conservative measures are implemented promptly. In contrast, advanced stages may necessitate more intensive interventions and carry different prognostic considerations. Prognostic information derived from staging enables clinicians to communicate effectively with patients and caregivers, setting realistic expectations regarding the potential outcomes of treatment. This informed approach enhances patient understanding and engagement in the decision-making process [20].

Monitoring disease progression: Staging facilitates systematic and longitudinal monitoring of disease progression in individuals with AVN. Periodic imaging assessments, guided by the staged classification, allow clinicians to track changes in the affected joint over time. This monitoring is particularly critical in managing chronic conditions like AVN, where disease progression may be gradual. Adjustments to treatment plans can be made based on observed changes, ensuring that interventions remain aligned with the evolving nature of the disease. Regular monitoring enhances the ability to detect subtle alterations in joint structure and function, enabling timely interventions to mitigate disease progression [21].

Research and clinical trials: Staging provides a standardized framework invaluable in research and clinical trials focused on AVN. Consistent staging criteria allow for accurately categorizing patients into specific disease stages, facilitating comparisons of treatment outcomes across diverse patient populations. The reliability and generalizability of research findings are enhanced through standardized staging, contributing to the development of evidence-based guidelines for AVN management. The systematic approach provided by staging criteria ensures that research outcomes apply to a broader patient demographic, fostering advancements in treatment strategies and contributing to the collective knowledge in the field [22].

Commonly Used Staging Systems

Several staging systems have been developed to classify AVN based on clinical and radiographic criteria. The choice of staging system may depend on the affected joint and the preferences of the treating physician. Some commonly used staging systems are described in Table 1.

**Table 1:** Circulation Osseous (ARCO) Staging for Hip AVN and Koo's Staging System for Knee AVN

Staging System	Stage	Description
Ficat and Arlet Classification (Hip AVN) [23]	I	Normal X-rays, but MRI may show early signs of AVN.
	II	X-rays reveal sclerosis and cyst formation without collapse.
	III	Crescent sign indicating subchondral collapse.
	IV	Joint space narrowing and degenerative changes.
Association Research Circulation Osseous (ARCO) Staging (Hip AVN) [24]	0	Normal X-rays, but MRI detects AVN.
	I	Normal X-rays, but early changes visible on MRI.
	II	Sclerosis or cyst formation without collapse.
	III	Subchondral collapse without joint space narrowing.
	IV	Joint space narrowing and secondary degenerative changes.
Koo's Staging System (Knee AVN) [25]	I	Normal X-rays but abnormal MRI findings.
	II	Sclerosis, cyst formation, or flattening without joint space narrowing.
	III	Joint space narrowing and subchondral collapse.
	IV	Advanced degenerative changes.

Conservative treatment approaches

Rest and Activity Modification

Protected weight-bearing: Implementing a strategy of protected weight-bearing is a fundamental component of the conservative treatment approach for individuals with AVN. This intervention aims to reduce mechanical stress on the affected joint, providing an environment conducive to healing compromised bone tissue. Restricting weight-bearing activities is achieved by advising individuals to minimize the use of the affected joint for bearing their body weight. Assistive devices such as crutches or walkers may be recommended to further alleviate the load on the compromised joint during activities like walking. This protective measure not only aids in pain management but also plays a crucial role in preventing further damage to the bone and promoting the potential for natural healing [26].

Activity modification: Activity modification is a crucial aspect of managing AVN, involving adjustments to daily activities to minimize strain on the affected joint. Individuals are guided to avoid activities that impose excessive stress on the compromised joint, such as prolonged standing or sitting, which could exacerbate symptoms and hinder healing. Moreover, limiting activities that involve repetitive joint movements is emphasized to prevent additional wear and tear on the affected area. Occupational and lifestyle adjustments are often tailored to the individual's specific condition, considering the location and severity of AVN. This personalized approach ensures that the activity modification plan aligns with the individual's daily routines and contributes to mitigating joint stress, managing symptoms, and supporting the healing process. The collaborative efforts of healthcare professionals, including orthopaedic specialists and physical therapists, are integral in guiding individuals through these modifications and optimizing their effectiveness for long-term joint health [9].

Pharmacological Interventions

Nonsteroidal anti-inflammatory drugs (NSAIDs): NSAIDs, including commonly used medications such as ibuprofen and naproxen, play a crucial role in the pharmacological management of AVN. These medications are employed to alleviate pain and reduce inflammation associated with the condition, providing symptomatic relief to individuals affected by AVN. By inhibiting the activity of enzymes responsible for producing inflammatory compounds, NSAIDs help mitigate pain and swelling in the affected joint. However, it is essential to note that the long-term use of NSAIDs may be associated with potential risks, particularly concerning gastrointestinal and cardiovascular health. Clinicians must carefully monitor patients using NSAIDs, considering individual health factors, pre-existing conditions, and the duration of treatment to minimize potential side effects and ensure the overall well-being of the patient [27].

Bisphosphonates: Bisphosphonates, which include medications such as alendronate and pamidronate, represent another class of drugs with potential implications for the management of AVN. These drugs are primarily known for preventing bone loss and maintaining bone density. In the context of AVN, bisphosphonates may slow the condition's progression by inhibiting bone resorption. By preserving bone structure, bisphosphonates aim to contribute to the overall management of AVN and potentially delay the need for surgical interventions. However, it is crucial to recognize that using bisphosphonates in AVN management is an area of ongoing research, and their effectiveness remains a subject of investigation. Furthermore, like any medication, bisphosphonates are associated with potential side effects, and their use should be carefully considered based on an individual's specific circumstances, including overall health, risk factors, and the stage of AVN. Regular monitoring and collaboration between healthcare professionals are essential to assess the benefits and risks of bisphosphonate therapy in AVN [28].

Physical Therapy

Range of motion exercises: Range of motion exercises are a cornerstone of the rehabilitation process for individuals with AVN. These gentle exercises are designed to maintain joint flexibility, prevent stiffness, and optimize overall joint function. Physical therapists are pivotal in developing individualized exercise programs tailored to each patient's needs and limitations. These programs may involve controlled movements that guide the affected joint through its full range, promoting joint health and preventing the development of contractures. Regularly implementing range of motion exercises contributes to the preservation of joint mobility and assists in mitigating the impact of AVN on daily activities [29].

Strengthening exercises: Targeted strengthening exercises form an integral part of rehabilitation for AVN, aiming to enhance the strength of the muscles surrounding the affected joint. By bolstering muscle support, these exercises help compensate for joint instability and reduce the overall load on the compromised bone. Strengthening programs address specific muscle groups related to the affected joint, promoting joint stability and optimizing functional capacity. Physical therapists work closely with patients to ensure that strengthening exercises are appropriately tailored, gradually progressing in intensity to avoid overexertion and accommodate the patient's capabilities [30].

Modalities for pain management: Pain management modalities are incorporated by physical therapists to alleviate discomfort associated with AVN and facilitate tissue healing. These may include heat or cold therapy, ultrasound, and electrical stimulation. Heat therapy can enhance blood flow, reduce muscle tension, and promote relaxation, while cold therapy can help alleviate inflammation and numb pain. Ultrasound and electrical stimulation may contribute to pain relief and tissue healing by affecting cellular function. The selection of specific modalities is based on the individual's needs, preferences, and stage of AVN, providing a comprehensive and personalized approach to pain management [31].

Functional training: Functional training is a critical component of rehabilitation for individuals with AVN, focusing on improving the patient's ability to perform daily activities with greater ease and efficiency. This form of training goes beyond isolated exercises and incorporates movements that mimic real-life activities. Physical therapists guide patients in practising proper body mechanics and joint protection techniques during functional tasks. By enhancing functional capacity, individuals can regain independence in their daily lives and minimize the impact of AVN on activities such as walking, reaching, and lifting. Functional training aims to optimize overall function and quality of life, promoting long-term joint health and mitigating the challenges posed by AVN [32].

Surgical treatment options

Surgical interventions play a crucial role in the management of AVN, especially when conservative measures fail to halt disease progression. The choice of surgical approach depends on factors such as the stage of AVN, the affected joint, and the patient's overall health. Several surgical treatment options are available, each addressing specific aspects of AVN.

Core Decompression

Technique: Core decompression is a surgical procedure for managing AVN. The technique involves making a small incision at the site of the affected joint, typically the femoral head, in the case of hip AVN. During the procedure, a core or plug of bone is carefully removed from the necrotic region. This deliberate removal of bone serves a dual purpose: first, it creates a void or channel within the affected area, and second, it facilitates the removal of the necrotic tissue. The goal is to create a pathway for new blood vessels to invade the necrotic region, potentially enhancing the blood supply to the compromised bone. Introducing fresh blood flow to the area is vital for promoting bone healing and regeneration. Core decompression is often considered in the early stages of AVN to intervene before extensive bone damage occurs. The success of this technique relies on the restoration of adequate blood circulation, contributing to the overall preservation of joint integrity [33].

Outcome: Core decompression has demonstrated success in clinical practice, offering relief from symptoms associated with AVN and, in some cases, slowing down the condition's progression. The procedure aims to provide a conducive environment for bone healing by creating a channel for improved blood supply. However, the effectiveness of core decompression can vary depending on factors such as the AVN stage, the affected joint's location, and the patient's overall health. In some instances, core decompression may be combined with additional therapeutic approaches to enhance its outcomes. This can include bone grafting, where healthy bone tissue is transplanted to the affected area to support regeneration further. The success of core decompression underscores the importance of early intervention and a multidisciplinary approach in the comprehensive management of AVN, with the ultimate goal of preserving joint function and improving the quality of life for affected individuals [34].

Vascularized Bone Grafting

Technique: Vascularized bone grafting is a surgical technique to treat AVN, particularly in advanced stages with extensive bone damage. The procedure involves harvesting a piece of bone, along with its accompanying blood vessels, from one area of the body and transplanting it to the site of the necrotic bone. The key distinction of vascularised bone grafting lies in preserving the blood supply to the transplanted bone. By ensuring the inclusion of blood vessels, the graft brings along a direct source of nourishment, facilitating the integration of the transplanted bone with the recipient site. This vascularised approach is particularly beneficial in cases where AVN has progressed, and there is a need for robust blood flow to support the healing and regeneration of the compromised bone. The technique is intricate and requires surgical expertise to carefully connect the blood vessels of the graft to those of the recipient site, ensuring optimal vascularisation and promoting successful graft integration [35].

Outcome: Vascularized bone grafting has demonstrated success in clinical practice, especially in cases with advanced stages of AVN characterized by extensive bone damage. Restoring a direct and immediate blood supply to the transplanted bone enhances the potential for successful healing and integration. This approach aims to preserve joint function by providing structural support to the compromised bone. While vascularised bone grafting is considered an effective intervention, it is more complex and involved than core decompression. As such, it is typically reserved for cases with significant bone involvement and when less invasive treatments may not be sufficient. The success of vascularised bone grafting underscores its role as a valuable option in the comprehensive management of AVN, particularly in situations where preserving joint integrity is critical for the patient's mobility and quality of life [36].

Total Joint Replacement

Indications: Total joint replacement, also known as arthroplasty, is a surgical intervention commonly indicated in cases of AVN with severe joint degeneration and functional impairment, particularly in weight-bearing joints such as the hip and knee. The decision to perform total joint replacement is typically made when conservative measures have proven ineffective, and the progression of AVN has resulted in significant damage to the joint structure. Indications for total joint replacement include persistent pain, loss of joint function, and a diminished quality of life due to the impact of AVN on daily activities. The procedure aims to alleviate pain, restore joint function, and enhance the overall mobility and well-being of the affected individual. Total joint replacement becomes a viable option when the joint damage is beyond the scope of other interventions, and the goal is to provide a durable and functional artificial joint that can mimic the natural joint's movement and stability [37].

Outcome: Total joint replacement has proven effective in providing long-term relief for individuals with end-stage AVN. Advances in prosthetic technology, surgical techniques, and postoperative care have contributed to improved outcomes, with many patients experiencing significant reductions in pain and restoration of functional capacity. The artificial joint, composed of metal, plastic, or ceramic materials, is designed to replicate the natural joint's movement and weight-bearing functions. Patients typically undergo extensive rehabilitation following the surgery to optimize recovery and regain joint function. The success of total joint replacement in AVN cases is reflected in improved quality of life, enhanced mobility, and a return to daily activities with reduced pain and increased joint stability. While the procedure is associated with a notable success rate, individual outcomes may vary, and careful consideration of factors such as patient health, age, and lifestyle is essential in the decision-making process. Overall, total joint replacement remains a critical and transformative intervention for individuals suffering from the debilitating effects of AVN in weight-bearing joints [38].

Arthroplasty Techniques

Hemiarthroplasty: Hemiarthroplasty is a surgical procedure in which only one part of the joint is replaced with a prosthetic component. This approach is commonly employed when only a specific portion of the joint is affected by AVN, and the remaining joint structures are relatively healthy. For example, in hip hemiarthroplasty, the femoral head, which may be the region impacted by AVN, is replaced with a prosthetic component. In contrast, the acetabulum (socket) of the hip joint remains intact. This technique addresses the localized damage within the joint, providing pain relief and restoring function without replacing the entire joint. Hemiarthroplasty is often considered in cases where the disease is asymmetrically distributed within the joint, and the preservation of native joint structures is deemed beneficial [39].

Resurfacing arthroplasty: Resurfacing arthroplasty involves removing and replacing the damaged surfaces of the joint while preserving more of the native bone compared to traditional joint replacement procedures. This technique is commonly employed in hip arthroplasty, specifically in cases where AVN affects the femoral head. In resurfacing, rather than entirely replacing the femoral head, the damaged surface is reshaped and capped with a metal prosthesis, preserving more of the patient's natural bone. This approach is designed to mimic the joint's natural anatomy more closely, potentially allowing for a more excellent range of motion and stability. Resurfacing arthroplasty is often considered in younger, more active patients, where preserving native bone may be advantageous for future revision surgeries [25].

Revision arthroplasty: Revision arthroplasty is a surgical procedure performed when previous joint replacement surgeries require correction, modification, or updating. This procedure involves removing and replacing existing prosthetic components to address complications, such as implant wear, loosening, or instability, and improve overall joint function. In the context of AVN, revision arthroplasty may be indicated if there are issues with the initially implanted joint replacement components or if the disease progresses following an earlier intervention. This complex procedure aims to rectify problems associated with the initial joint replacement, often involving new or revised prosthetic components to optimize joint stability and function. Revision arthroplasty requires careful planning and expertise to achieve successful outcomes and may involve more extensive surgical manoeuvres than primary joint replacement procedures [40].

Emerging therapies and research

Regenerative Medicine

Platelet-rich plasma (PRP): PRP is a regenerative therapy that involves the extraction and concentration of platelets from the patient's blood. The process begins with collecting a blood sample, which is then processed to separate the platelets from other blood components. The resulting platelet-rich plasma, abundant in growth factors, is injected into the affected joint. The high concentration of growth factors in PRP is believed to stimulate tissue repair and regeneration. In the context of AVN, PRP holds promise as a potential treatment option. Research is ongoing to explore its efficacy in promoting angiogenesis (forming new blood vessels) and reducing inflammation within the affected joint. By harnessing the body's natural healing mechanisms, PRP aims to improve blood flow to the necrotic area and create an environment conducive to tissue healing [41].

Bone marrow aspirate concentrate (BMAC): BMAC is a regenerative therapy that involves the extraction of bone marrow from the patient and the subsequent concentration of mesenchymal stem cells and growth factors. Like PRP, the process begins with collecting a bone marrow sample, typically from the patient's hip or pelvis. The harvested material is then processed to concentrate the mesenchymal stem cells and growth factors before being injected into the affected joint. BMAC aims to enhance tissue repair and promote bone regeneration in conditions such as AVN. Early studies suggest potential benefits, particularly in the early stages of AVN, where the goal is to intervene before extensive bone damage occurs. The concentrated solution, rich in regenerative components, is believed to contribute to the repair of the necrotic bone and the restoration of joint function. While research on the efficacy of BMAC in AVN is ongoing, the therapy represents a promising avenue in regenerative medicine for addressing the challenges posed by this condition [42].

Stem Cell Therapy

Mesenchymal stem cells (MSCs): MSCs, derived from sources such as bone marrow or adipose tissue, have emerged as a promising avenue in regenerative medicine for the treatment of AVN. MSCs possess the remarkable ability to differentiate into various cell types, including bone-forming cells (osteoblasts). In the context of AVN, where compromised blood supply leads to bone tissue death, MSCs offer a potential solution. Preclinical studies and early clinical trials have explored using MSCs to promote bone regeneration and repair necrotic tissue within the affected joint. The idea is that introducing MSCs to the necrotic area may enhance the body's natural healing processes, contributing to the formation of new, healthy bones. While research is ongoing, the regenerative potential of MSCs makes them a compelling candidate for future therapeutic applications in AVN [43].

Embryonic stem cells (ESCs) and induced pluripotent stem cells (iPSCs): Research on the use of pluripotent stem cells, including ESCs and iPSCs, for the treatment of AVN, is still in the experimental stages. ESCs are derived from embryos, and iPSCs are generated by reprogramming adult cells to a pluripotent state. Both pluripotent stem cells can differentiate into various cell types, including bone-forming cells (osteoblasts). This unique ability to become various cell types positions pluripotent stem cells as potentially transformative in regenerative therapies for conditions like AVN. While the research is in its early phases, the concept involves directing these cells to differentiate into bone-forming cells, providing a targeted approach to regenerate the necrotic bone tissue. Challenges related to safety, ethical considerations, and the optimization of differentiation protocols are essential aspects of ongoing research. The potential of pluripotent stem cells in AVN treatment opens avenues for innovative regenerative strategies, but their clinical application is likely to evolve as research progresses and addresses existing challenges [44].

Novel Pharmacological Approaches

Angiogenesis modulators: Angiogenesis modulators are medications currently being investigated for their potential in treating AVN. These agents aim to promote angiogenesis, which is the formation of new blood vessels. In the context of AVN, where compromised blood supply leads to ischemia and necrosis of bone tissue, promoting the growth of new blood vessels holds promise for improving blood flow to the affected area. By enhancing angiogenesis, these medications seek to create a more favourable environment for tissue healing and regeneration. Research is ongoing to assess the effectiveness of angiogenesis modulators in mitigating the ischemic effects of AVN and potentially slowing disease progression [45].

Bone-targeted therapies: Novel pharmacological approaches in AVN treatment focus on bone-targeted therapies that specifically address bone remodelling and repair pathways. These drugs aim to modulate bone metabolism and enhance bone formation, offering a targeted approach to address the underlying pathology of AVN. The goal is to support the regeneration of necrotic bone tissue and potentially prevent further deterioration. As research progresses, the development of bone-targeted therapies may provide additional options for managing AVN, especially in cases where the disease has not progressed to advanced stages [46].

Anti-inflammatory agents: Recognizing the inflammatory component of AVN, new anti-inflammatory medications are being explored as potential interventions. These drugs aim to reduce inflammation within the affected joint, potentially alleviating symptoms and slowing disease progression. In AVN, inflammation plays a role in the pathogenesis and contributes to the destruction of bone tissue. Therefore, anti-inflammatory agents may offer a complementary approach to managing the condition, particularly with other treatment modalities. Ongoing research aims to evaluate the efficacy of these medications in addressing the inflammatory aspects of AVN and their potential role in a comprehensive treatment strategy [47].

Rehabilitation and post-treatment care

Physical Therapy Protocols

Range of motion exercises: Range of motion exercises play a pivotal role in the early stages of rehabilitation, aiming to improve joint flexibility and prevent stiffness. These exercises involve controlled movements that guide the affected joint through its full range of motion. By promoting flexibility, range of motion exercises restore normal joint movement, reduce the risk of contractures, and optimize overall joint function [48].

Strengthening exercises: Progressive strengthening exercises form a crucial component of rehabilitation, explicitly targeting the muscles around the affected joint. These exercises enhance muscle strength, providing stability and support to the treated joint. Strengthening the muscles becomes particularly important in cases where joint instability or muscle atrophy has occurred due to conditions like AVN. The gradual progression of strengthening exercises contributes to overall rehabilitation, promoting joint stability and preventing further functional decline [49].

Weight-bearing activities: The reintroduction of weight-bearing activities is a carefully monitored aspect of rehabilitation, aiming to avoid excessive stress on the treated joint. This phase involves a gradual progression of weight-bearing exercises, starting with low-impact activities and advancing to more demanding tasks as tolerated by the patient. The controlled reintroduction of weight-bearing helps assess the joint's response to increased load, ensuring a safe and effective rehabilitation process while minimizing the risk of complications [50].

Functional training: Functional training is a targeted approach in rehabilitation, focusing on enhancing the patient's ability to perform daily activities with improved efficiency and reduced strain on the treated joint. This form of training goes beyond isolated exercises and incorporates movements that mimic real-life activities. Occupational considerations and biomechanical principles are integrated to ensure the patient can safely and effectively engage in functional tasks, promoting independence and return to daily activities [51].

Pain management techniques: Physical therapists employ various pain management modalities as part of rehabilitation to alleviate discomfort and enhance the patient's ability to engage in exercises. Modalities such as heat or cold therapy, massage, and transcutaneous electrical nerve stimulation (TENS) address pain and discomfort associated with the treated joint. Integrating pain management techniques aims to create a more comfortable and conducive environment for the patient to participate actively in the rehabilitation process [52].

Follow-up Monitoring

Imaging studies: Regular imaging studies, such as X-rays or MRI, are pivotal in post-treatment care to assess the healing progress and identify potential complications. These studies allow healthcare providers to monitor changes in bone structure and joint integrity, providing valuable insights into the effectiveness of the treatment. Periodic imaging helps detect any signs of relapse early, ensuring timely intervention and adjustment of the patient's care plan [53].

Clinical assessment: Ongoing clinical assessments are fundamental in post-treatment care, encompassing evaluations of joint function, range of motion, and strength. These assessments are essential for tracking the patient's rehabilitation progress and identifying areas that may require further attention or modification in the rehabilitation plan. Clinicians use hands-on evaluations and functional tests to gauge the individual's response to treatment, enabling them to tailor rehabilitation strategies to each patient's specific needs and challenges [54].

Pain assessment: Periodic pain levels and symptomatology assessment are critical in post-treatment care. Monitoring changes in pain patterns or intensity provides essential feedback on the patient's response to rehabilitation and the overall healing process. Pain assessment guides healthcare providers in making informed decisions regarding the continuation of specific exercises, the need for adjustments in the rehabilitation plan, or the consideration of additional medical interventions to manage pain effectively. A comprehensive understanding of the patient's pain experience is integral to optimizing post-treatment care and ensuring a successful recovery [55].

Long-term Management Strategies

Lifestyle modifications: Lifestyle modifications play a crucial role in managing AVN in the long term and aim to reduce stress on the affected joint. Patients are often advised to use weight management strategies, as excess body weight can increase joint stress. Adopting joint-friendly exercise routines, such as low-impact activities, can help maintain joint health without exacerbating symptoms. Lifestyle modifications are tailored to the individual's needs and are designed to promote overall joint well-being while minimizing the risk of further damage [56].

Joint protection techniques: Educating individuals on joint protection techniques is integral to managing AVN. These techniques empower patients to optimize their daily activities without putting undue stress on the affected joint. Proper body mechanics, ergonomic adjustments in work and daily environments, and adaptive devices, when necessary, contribute to joint protection. By incorporating these techniques into daily routines, individuals with AVN can enhance their ability to perform activities while minimizing the risk of exacerbating joint symptoms [57].

Medication management: Long-term pharmacological management is often necessary to address pain and inflammation associated with AVN. Medications such as analgesics (pain relievers) or disease-modifying anti-rheumatic drugs (DMARDs) may be prescribed based on individual needs. Analgesics help manage pain symptoms, while DMARDs may be used to modify the disease process in cases where AVN is associated with autoimmune or inflammatory conditions. Medication management is tailored to the specific requirements of each patient and is an essential component of comprehensive AVN care [58].

Regular follow-up with healthcare providers: Regular follow-up appointments with orthopaedic specialists or rheumatologists are essential for ongoing joint health monitoring in individuals with AVN. These appointments allow for continuous assessment of the joint's condition and the early detection of any recurrent symptoms or signs of disease progression. This proactive approach enables healthcare providers to intervene promptly, adjusting treatment plans or recommending additional interventions to prevent further joint deterioration [59].

Patient education and empowerment: Patient education is a cornerstone of effective AVN management, promoting active participation in maintaining joint health. Empowering patients with knowledge about their condition, treatment options, and self-management strategies is vital. Education covers the importance of adherence to rehabilitation protocols, the implementation of lifestyle modifications, and the significance of regular follow-up care. By understanding their condition and actively participating in their care, patients can make informed decisions and take proactive steps to preserve joint function and overall well-being. Patient empowerment fosters a collaborative approach between healthcare providers and individuals, optimizing the long-term management of AVN [60].

Challenges and future directions

Limitations of Current Treatment Strategies

Stage-specific interventions: The current landscape of AVN treatment strategies recognizes the importance of stage-specific interventions, tailoring approaches based on the severity of the disease. While more established interventions exist for early-stage AVN, challenges persist in addressing advanced-stage cases, especially in weight-bearing joints. Research advances are crucial to developing effective interventions for late-stage disease, focusing on preserving joint function, alleviating symptoms, and preventing further degeneration. The refinement and expansion of treatment options for advanced-stage AVN remain areas of active investigation to enhance patient outcomes [9].

Risk of complications: Although effective in many instances, surgical interventions have inherent risks and potential complications. Complications such as infection, implant failure, or incomplete resolution of symptoms underscore the need for continuous improvement in surgical techniques and postoperative care. Research and innovation in the field aim to minimize these risks, enhance the safety profile of surgical interventions, and optimize the overall success of treatment. Ongoing efforts focus on refining surgical approaches and developing strategies to mitigate the potential complications associated with AVN interventions [61].

Limited pharmacological options: Current pharmacological interventions, including bisphosphonates and NSAIDs, primarily provide symptomatic relief and may not address the underlying causes of AVN. The quest for more targeted pharmacological agents is a crucial focus of ongoing research. Developing medications that alleviate symptoms and promote bone regeneration and vascular health is a critical area of exploration. Advancements in pharmacological options aim to offer more comprehensive and disease-modifying treatments, addressing the root causes of AVN and improving long-term outcomes for affected individuals [27].

Heterogeneity in patient response: The heterogeneity in patient response to conservative and surgical treatments poses a complex challenge in AVN management. Variability in treatment outcomes among individuals underscores the importance of identifying predictive factors influencing responses to different interventions. Personalized medicine approaches, guided by patient-specific characteristics, genetic factors, and disease mechanisms, are under investigation to tailor interventions to individual profiles. Understanding the factors contributing to diverse treatment responses is essential for refining treatment algorithms and optimizing the effectiveness of interventions for each patient with AVN. Ongoing research explores the complexities of individualized treatment responses in this multifaceted condition [62].

Areas for Further Research

Biomarkers for early detection: Advancing research should prioritize the identification of reliable biomarkers for the early detection of AVN. Biomarkers that signal a predisposition to AVN or indicate early vascular changes could revolutionize clinical practice by enabling timely intervention and preventive strategies. Developing robust and sensitive biomarkers can transform AVN diagnosis and management, allowing healthcare providers to identify at-risk individuals and implement targeted interventions before irreversible damage occurs [63].

Genetic and molecular pathways: A deeper exploration of the genetic and molecular pathways involved in AVN development is crucial for gaining insights into targeted therapeutic approaches. Investigating the genetic factors contributing to AVN susceptibility provides an avenue for personalized treatment strategies. Understanding the intricate molecular mechanisms underlying AVN pathogenesis could lead to developing interventions that address the condition's root causes. Research in this area has the potential to unlock new therapeutic targets and guide the development of precision medicine approaches tailored to individual patients [64].

Regenerative therapies: Further research into regenerative therapies, including stem cell therapy and tissue engineering, is essential for advancing treatment options for AVN. Enhancing the regenerative potential of these therapies and optimizing their application for different joints and AVN stages is an ongoing exploration area. Investigating the mechanisms by which regenerative therapies promote tissue repair and exploring their long-term efficacy will contribute to the development of innovative and sustainable interventions for AVN [65].

Improved imaging techniques: Advancements in imaging techniques, particularly advanced MRI and molecular imaging, are crucial for enhancing our ability to visualize early changes in AVN bone vascularity and tissue health. Improved imaging modalities can provide more accurate and detailed information, enabling precise diagnosis and staging of AVN. Early vascular and structural changes detection through advanced imaging can facilitate timely intervention and guide treatment decisions. Research efforts focused on refining and developing imaging technologies will improve diagnostic capabilities and AVN management [66].

Integrating Multidisciplinary Approaches

Collaboration among specialities: Effective AVN management necessitates seamless collaboration among various specialities, including orthopaedic surgeons, rheumatologists, radiologists, and physical therapists. Establishing multidisciplinary clinics where specialists work collaboratively ensures that individuals with AVN receive comprehensive and coordinated care. This collaborative approach enables the integration of diverse expertise, fostering a holistic understanding of the condition and optimizing treatment strategies tailored to each patient's needs [67].

Patient-centred care: Embracing a patient-centred approach is integral to AVN care, emphasizing the importance of considering individual preferences, lifestyles, and treatment goals. Incorporating patient perspectives into treatment decision-making and care planning enhances treatment adherence and satisfaction. By actively involving patients in their care, healthcare providers can build a partnership that empowers individuals to participate in their treatment journey, leading to more personalized and effective interventions [68].

Rehabilitation and lifestyle interventions: Integration of lifestyle interventions, including weight management, joint protection strategies, and patient education, is critical for achieving long-term success in AVN management. Multidisciplinary teams can collaborate to develop comprehensive rehabilitation and lifestyle programs tailored to individual needs. This collaborative effort addresses not only the immediate symptoms but also focuses on enhancing overall joint health and minimizing the risk of disease progression through proactive lifestyle modifications [69].

Telemedicine and remote monitoring: Exploring telemedicine and remote monitoring technologies represents a promising avenue to facilitate ongoing care and follow-up for individuals with AVN. Especially beneficial for patients in remote locations or those with mobility limitations, these technologies can enhance accessibility to multidisciplinary care. Telemedicine enables virtual consultations, allowing patients to connect with specialists and receive timely guidance. Remote monitoring tools can track key indicators of joint health, providing valuable data for healthcare providers to assess treatment effectiveness and make informed adjustments, thereby ensuring continuous and responsive care [70].

## Conclusions

In conclusion, AVN presents a multifaceted challenge in orthopaedic and rheumatologic practice. This comprehensive review has highlighted vital findings, emphasizing the importance of early detection and a multidisciplinary approach to care. From recognizing aetiological factors and clinical presentations to exploring conservative and surgical interventions, the varied nature of AVN requires individualized treatment plans. Emerging therapies, including regenerative medicine and stem cell therapy, offer promising avenues for future exploration alongside ongoing research into genetic and molecular pathways. The implications for clinical practice stress the need for personalized care, staying informed about advancements, and incorporating patient education. Recommendations for future management strategies underscore the importance of advancing imaging technology, integrating regenerative therapies, and empowering patients in their healthcare journey. As we navigate the complexities of AVN, translating research findings into clinical practice holds the key to improving outcomes and enhancing the overall quality of life for those affected by this challenging condition.
